# Publisher Correction: Integrating chemical and mechanical signals through dynamic coupling between cellular protrusions and pulsed ERK activation

**DOI:** 10.1038/s41467-019-08341-8

**Published:** 2019-01-15

**Authors:** Jr-Ming Yang, Sayak Bhattacharya, Hoku West-Foyle, Chien-Fu Hung, T.-C. Wu, Pablo A. Iglesias, Chuan-Hsiang Huang

**Affiliations:** 1 Department of Pathology, Johns Hopkins Medical Institutions, Baltimore, MD 21205 USA; 2 Department of Electrical and Computer Engineering, Whiting School of Engineering, Johns Hopkins University, Baltimore, MD 21218 USA; 3 Department of Cell Biology, Johns Hopkins School of Medicine, Baltimore, MD 21205 USA; 4 Department of Oncology, Johns Hopkins Medical Institutions, Baltimore, MD 21205 USA; 5 Department of Obstetrics and Gynecology, Johns Hopkins Medical Institutions, Baltimore, MD 21205 USA; 6 Department of Molecular Microbiology and Immunology, Johns Hopkins Medical Institutions, Baltimore, MD 21205 USA

Correction to: *Nature Communications* 10.1038/s41467-018-07150-9; published online 07 November 2018

In the original version of this Article, the label “RTK” in Fig. 6a was inadvertently changed to “RTE”. This has now been corrected in the PDF and HTML versions of the Article.

