# Publisher Correction: Integrating chemical and mechanical signals through dynamic coupling between cellular protrusions and pulsed ERK activation

**DOI:** 10.1038/s41467-019-08330-x

**Published:** 2019-01-15

**Authors:** Jr-Ming Yang, Sayak Bhattacharya, Hoku West-Foyle, Chien-Fu Hung, T.-C. Wu, Pablo A. Iglesias, Chuan-Hsiang Huang

**Affiliations:** 1 Department of Pathology, Johns Hopkins Medical Institutions, Baltimore, MD 21205 USA; 2 Department of Electrical and Computer Engineering, Whiting School of Engineering, Johns Hopkins University, Baltimore, MD 21218 USA; 3 Department of Cell Biology, Johns Hopkins School of Medicine, Baltimore, MD 21205 USA; 4 Department of Oncology, Johns Hopkins Medical Institutions, Baltimore, MD 21205 USA; 5 Department of Obstetrics and Gynecology, Johns Hopkins Medical Institutions, Baltimore, MD 21205 USA; 6 Department of Molecular Microbiology and Immunology, Johns Hopkins Medical Institutions, Baltimore, MD 21205 USA

Correction to: *Nature Communications*; 10.1038/s41467-018-07150-9; published online: 07 November 2018

The original version of this Article contained an error in the spelling of the author Jr-Ming Yang, which was incorrectly given as J.-Ming Yang. This has now been corrected in both the PDF and HTML versions of the Article.

