# Supplementary material for: Integrating chemical and mechanical signals through dynamic coupling between cellular protrusions and pulsed ERK activation
Source: Nat Commun. 2018 Nov 7;9:4673. doi: 10.1038/s41467-018-07150-9 (PMC6220176; doi:10.1038/s41467-018-07150-9)
Supplement: Supplementary file 1 — Supplementary Information [file 41467_2018_7150_MOESM1_ESM.pdf]

Supplementary Information:

Integrating chemical and mechanical signals through dynamic coupling between cellular protrusions and pulsed ERK activation

Yang et al.

Supplementary Figure 1

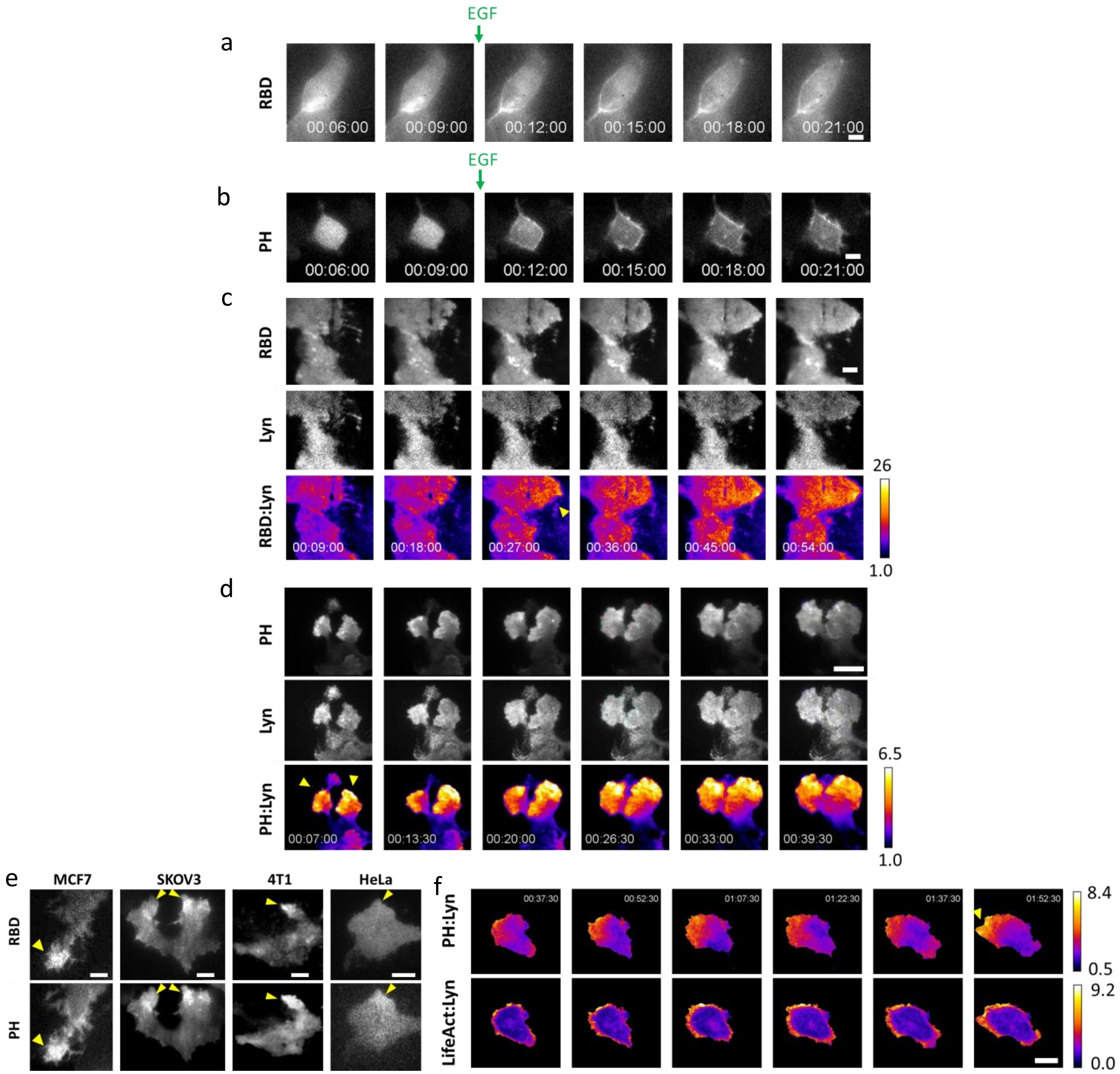

Supplementary Figure 1. Ras, PI3K, and cytoskeletal activities on protrusions. (a-b) Time-lapse epifluorescence images of MCF7 cells showing membrane recruitment of RBD (a) and PH-AKT (b) in response to EGF stimulation. (c) Ras activation on spontaneous protrusions revealed by time-lapse TIRF images of an SKOV3 cell expressing RBD-GFP and the membrane marker Lyn-CFP (corresponding to Supplementary Movie 1). The RBD:Lyn ratio was increased at the protrusion (arrowhead). Similarly, PI3K activation on spontaneous protrusions was shown by TIRF images of a cell expressing PH-AKT-RFP and Lyn-CFP (d, corresponding to Supplementary Movie 2). (e) Colocalization of RBD-GFP and PH-AKT-RFP on protrusions (arrowheads) in different cell lines. (f) Time-lapse TIRF images of an SKOV3 cell co-expressing PH-AKT-GFP, LifeAct-RFP, and Lyn-CFP showing increased PH-AKT:Lyn and LifeAct:Lyn on the protrusion (arrowhead). Cells also make undulations along the perimeter enriched for LifeAct but not PH-AKT (see Supplementary Movie 3). Scale bars: 10 µm.

Supplementary Figure 2

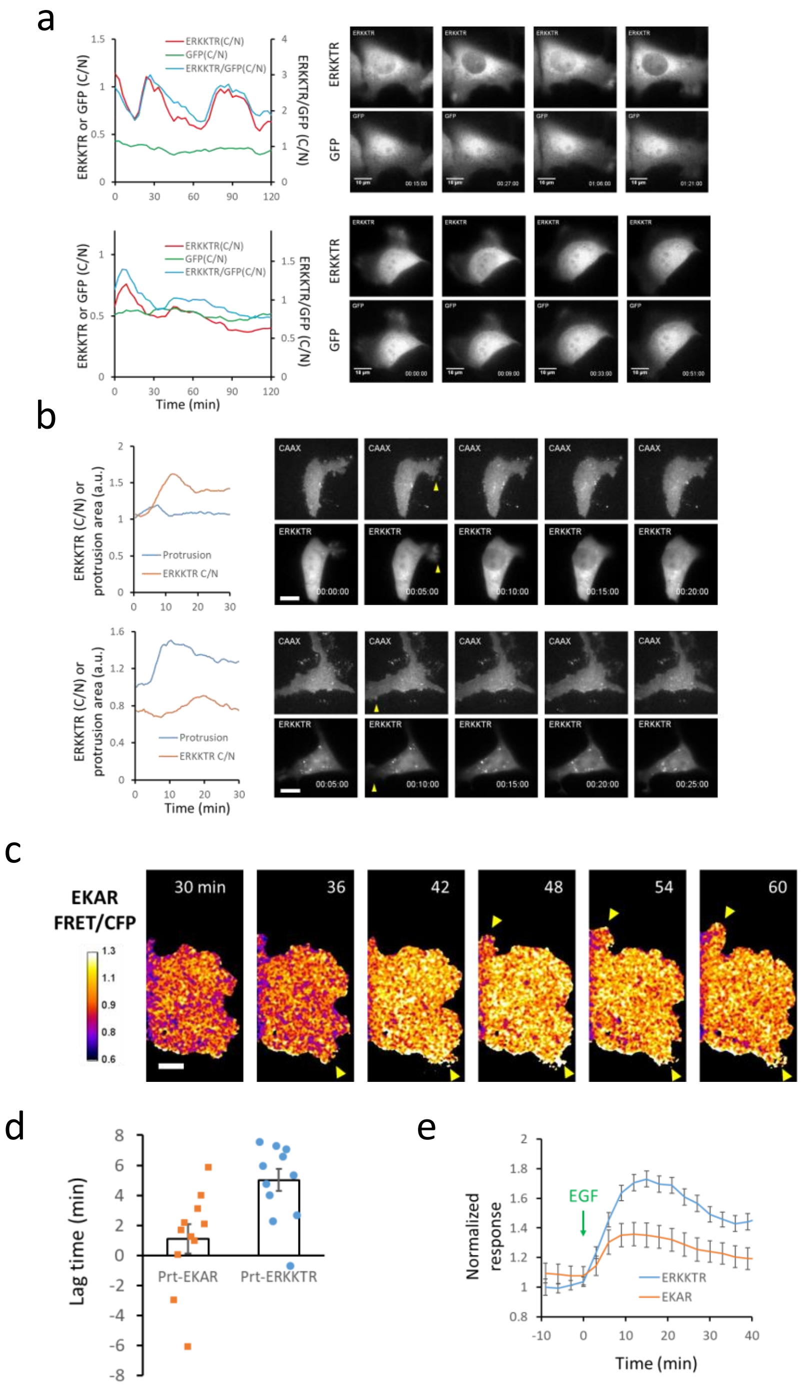

Supplementary Figure 2. Biosensor characterization. (a) Time-lapse epifluorescence images of two MCF7 cells co-expressing ERKKTR-RFP and GFP along with temporal profile of cytosolic-to-nuclear (C/N) ratios of ERKKTR, GFP, and ERKKTR normalized to GFP. (b) Time-lapse images of two MCF7 cells co-expressing ERKKTR-RFP (epifluorescence) and a CAAX membrane marker (TIRF) along with temporal profiles of ERKKTR (C/N) and normalized protrusion area. (c) TIRF images of an MCF7 cell expressing EKAR, a FRET reporter for ERK activation. Arrowheads indicate protrusions. (d) Quantification of lag time between half-maximal levels of protrusion area and those of EKAR FRET or ERKKTR C/N ratios (mean ± s.e.m., n=12 for EKAR and 11 for ERKKTR). (e) Kinetics of ERKKTR (normalized C/N ratio) and EKAR (FRET/CFP) responding to 20 ng/ml EGF stimulation (mean ± s.e.m., n=14 for EKAR, 57 for ERKKTR). Scale bars: 10 µm.

Supplementary Figure 3

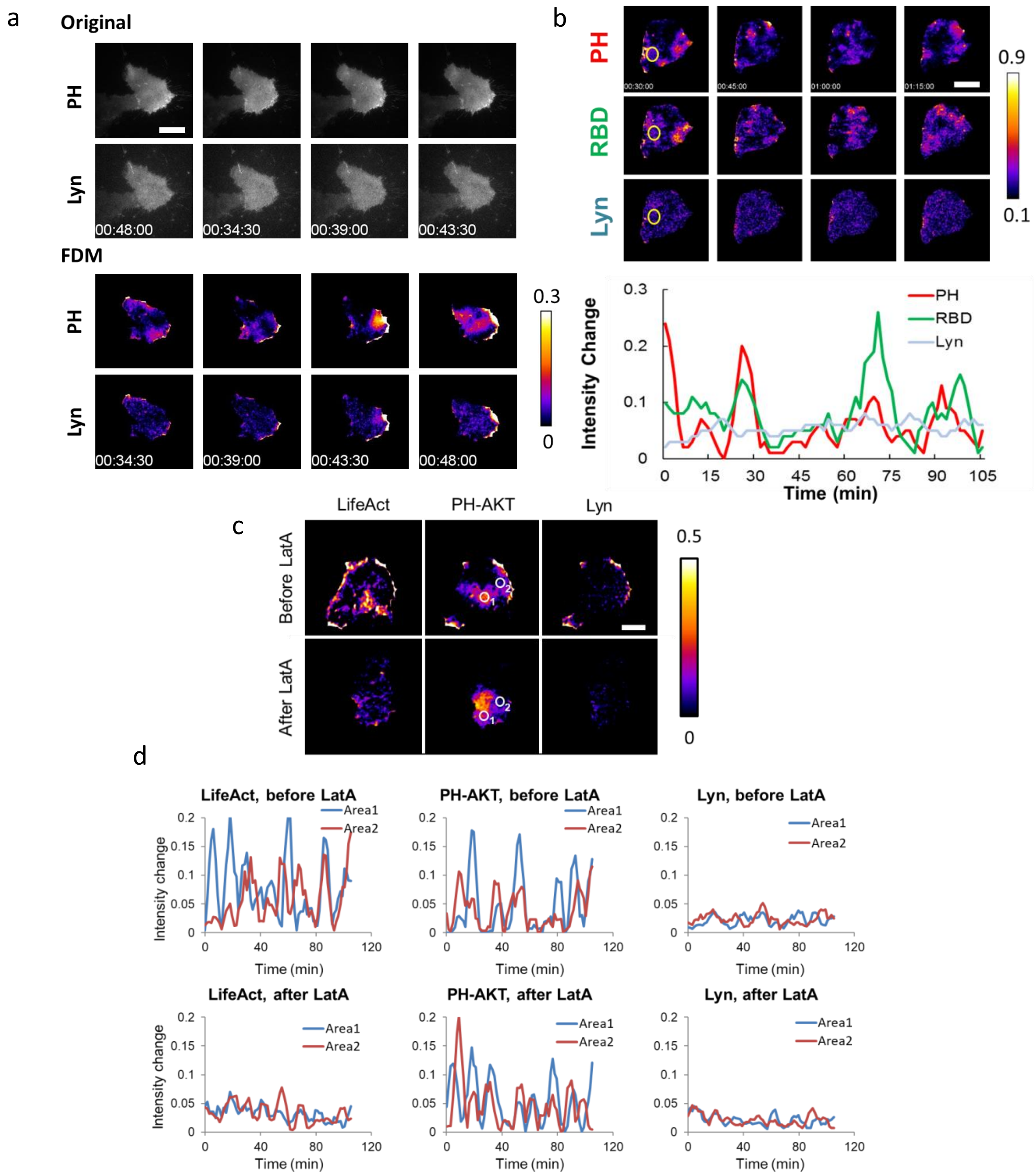

Supplementary Figure 3. Cytoskeleton-independent activities of Ras and PI3K in the interior region of basal cell surface. (a) Time-lapse TIRF images of an SKOV3 cell showing internal flashes of PH-AKT but not Lyn (membrane marker). The temporal changes in PH-AKT fluorescence is demonstrated by the Frame Difference Method (FDM, see Methods). (b) (Top) FDM analysis for PH-AKT, RBD, and Lyn. (Bottom) Plot of the intensity of the biosensors over the circular region revealed synchronized Ras and PI3K activation. (c) FDM analysis for an SKOV3 cell expressing LifeAct-RFP, PH-AKT-GFP, and Lyn-CFP showing persistence of Internal flashes of PH-AKT after addition of latrunculin (corresponding to Supplementary Movie 6). In contrast, flashes of LifeAct was inhibited by latrunculin. The temporal profile for two circular areas labeled 1 and 2 is shown in (d). Scale bars: 10 μm.

Supplementary Figure 4

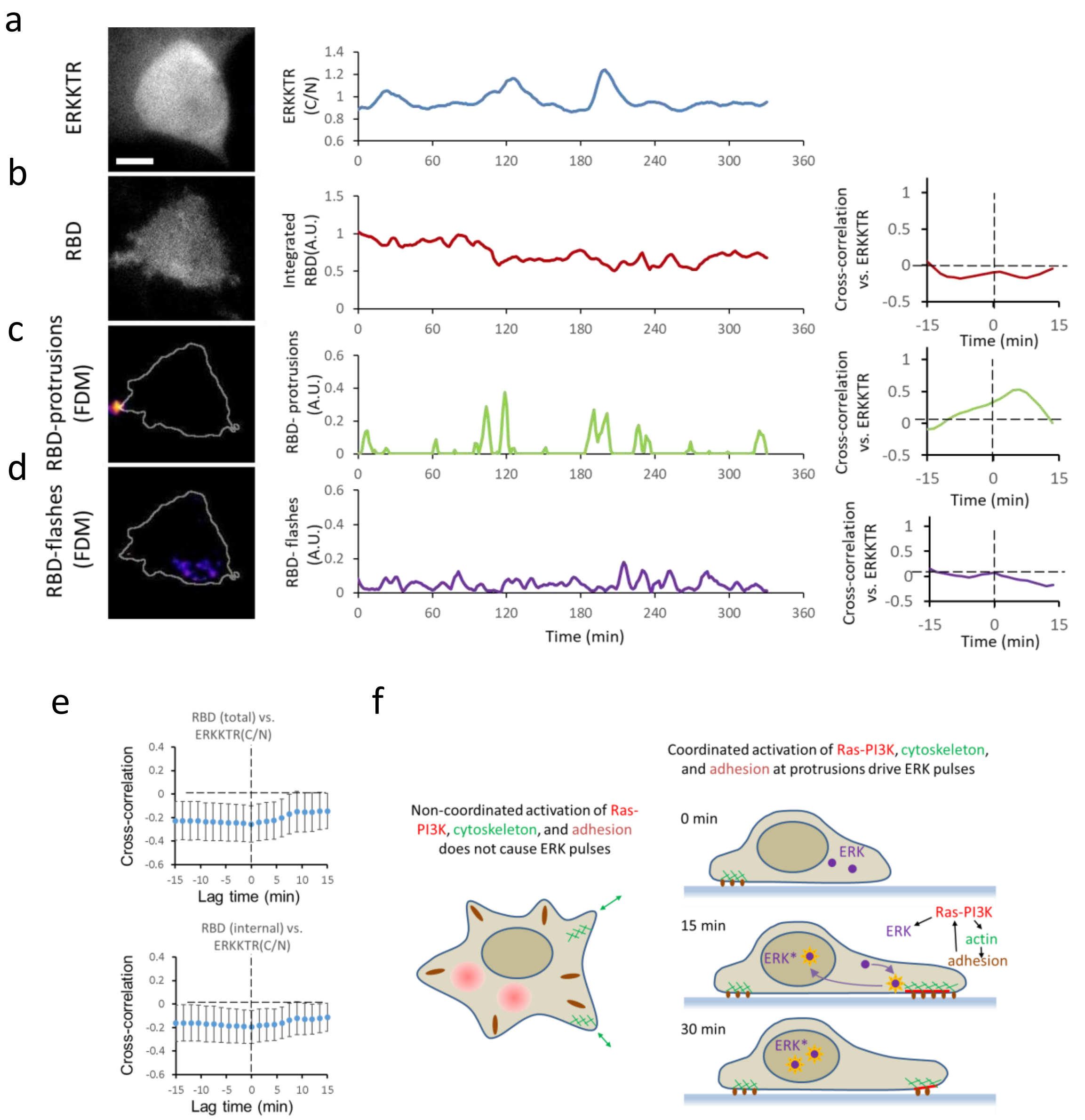

Supplementary Figure 4. ERK activation does not correlate with internal flashes or integrated activities of Ras. Temporal profiles of (a) ERKKTR (C/N), (b) total RBD, (c) integrated RBD on protrusions, and (d) integrated RBD in internal flashes of an MCF7 cell (the same cell as in Fig 1g and Supplementary Movie 4). The RBD-enriched protrusions (c) and internal flashes (d) were identified by FDM (see Methods). Cross-correlation of RBD vs. ERKKTR is shown on the right. Scale bar: 10 µm. (e) Cross-correlation analysis of total and internal RBD vs. ERKKTR (mean ± s.e.m., n=9 videos). (f) Cartoon of ERK activation driven by spatiotemporally coordinated Ras-PI3K, cytoskeletal, adhesion activities in protrusions but not by uncoordinated activities.

Supplementary Figure 5

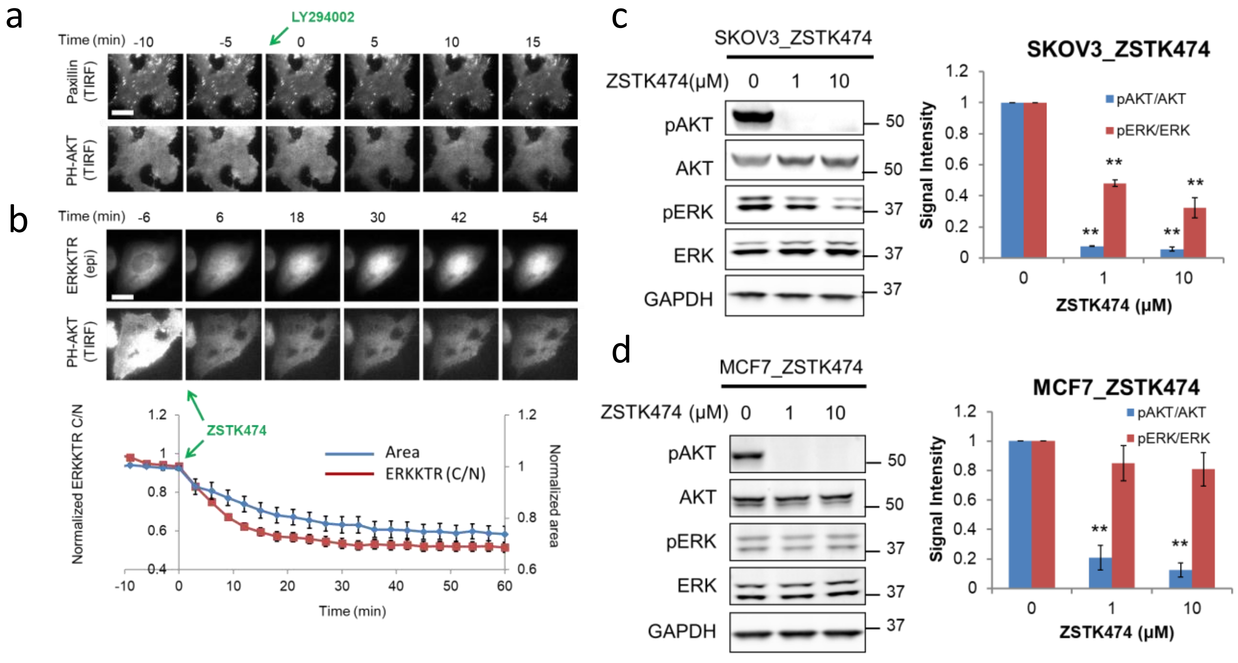

Supplementary Figure 5. Effects of perturbations to Ras-PI3K signaling. (a-b) Time-lapse images of SKOV3 cells showing loss of paxillin (a) as well as nuclear entry of ERKKTR and cell area change (b) upon PI3K inhibition by 50 µM LY294002 (a) or 10 µM ZSTK474 (b). The kinetics of ERKKTR nuclear translocation as well as the attached cell surface area upon PI3K inhibition is also shown (b, bottom, n=13 and 14 cells for ERKKTR and area, respectively; error bar: s.e.m.). (c-d) Immunoblot of phospho-AKT and phospho-ERK in SKOV3 (c) and MCF7 (d) cells treated with 0 (DMSO control), 1 and 10 µM ZSTK474. Quantification is shown on the right (n=3, error bar: s.e.m.) * p<0.05; ** p<0.005 ** by two-tail unpaired t test. Scale bars: 10 µm.

Supplementary Figure 6

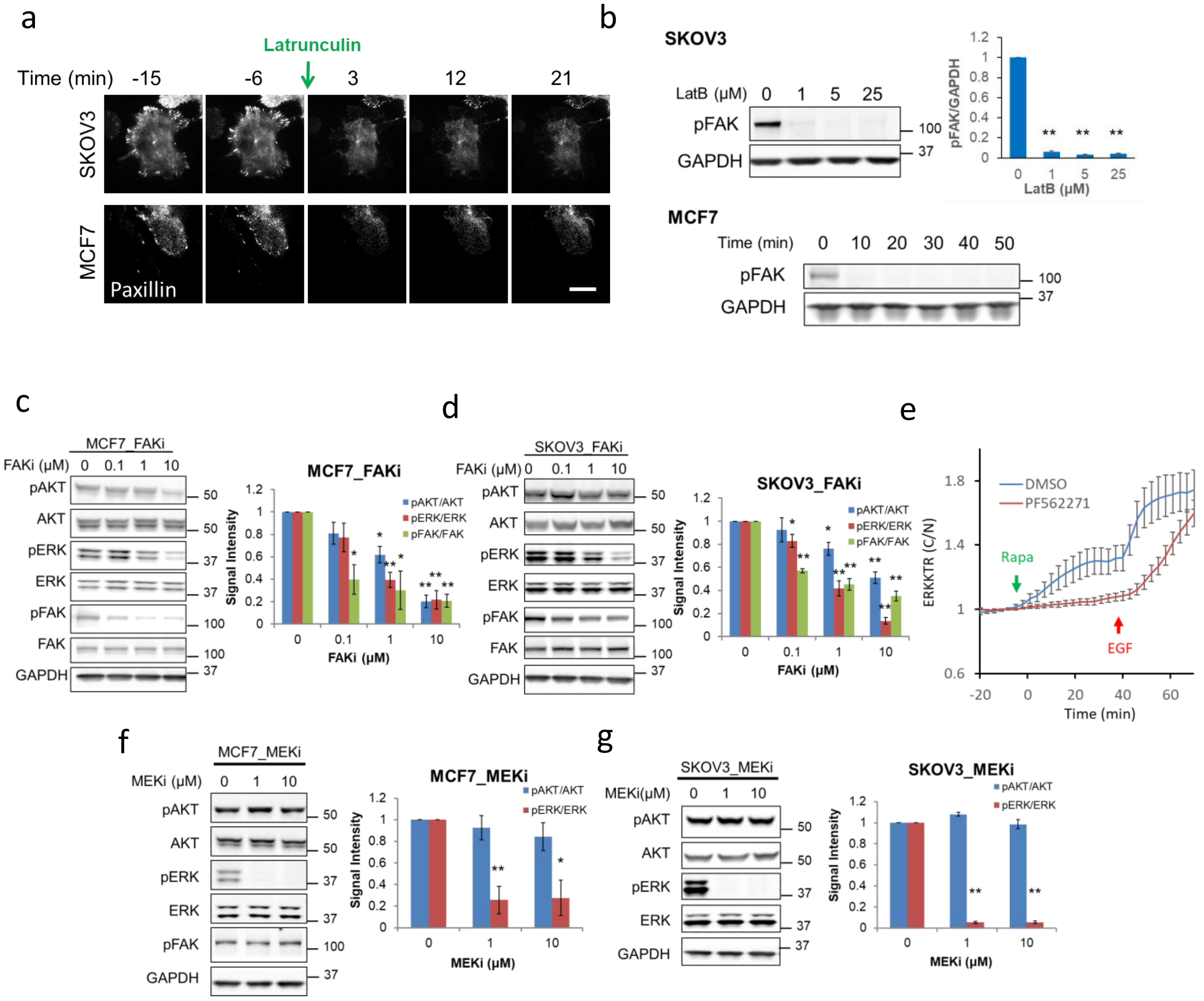

Supplementary Figure 6. Effects of perturbations to ERK, cytoskeleton, and adhesion. (a) Time-lapse images of SKOV3 and MCF7 cells showing loss of paxillin patches upon latrunculin treatment. Scale bar: 10 µm. (b) Latrunculin B dose curve for phospho-FAK in SKOV3 cells (top, N=4) and time course of phospho-FAK in response to latrunculin in MCF7 (bottom). (c-d) Immunoblot of phospho-AKT, phospho-ERK, and phospho-FAK for MCF7 cells (c) and SKOV3 cells (d) treated with the FAK inhibitor PF-573228 for an hour. (e) Response to CID of Tiam1 in HeLa cells pre-treated with DMSO or 10 µM FAK inhibitor PF-562271 for 1 hour (mean ± s.e.m., n=27 and 28 cells for DMSO and PF-562271, respectively). EGF (100 ng/mL) stimulation was given at the indicated time point (red arrow). See Fig. 4g for a similar experiment using the FAK inhibitor PF-573228. (f-g) Immunoblot of phospho-AKT and phospho-ERK for MCF7 cells (e) and SKOV3 (f) cells treated with 0 (DMSO control), 1 and 10 µM MEK inhibitor PD325901 for an hour**.** N=3 unless otherwise indicated. Error bars: s.e.m. * p<0.05; ** p<0.005 by two-tail unpaired t test.

Supplementary Figure 7

Uncropped immunoblots for Fig. 2b

pERK

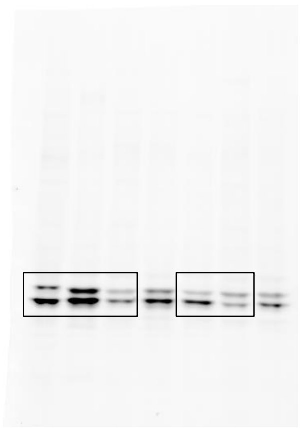

pERK long exposure

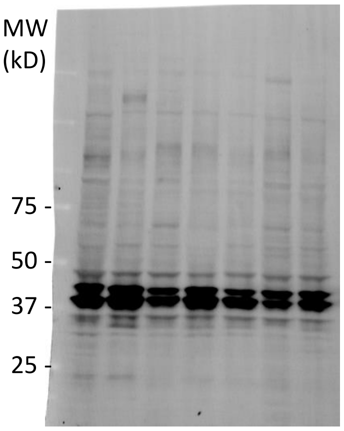

GAPDH

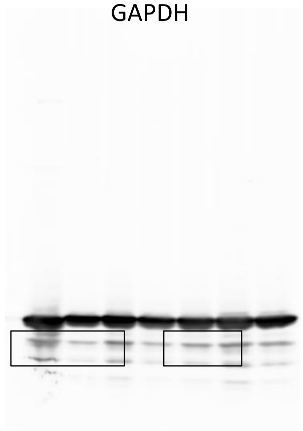

GAPDH long exposure

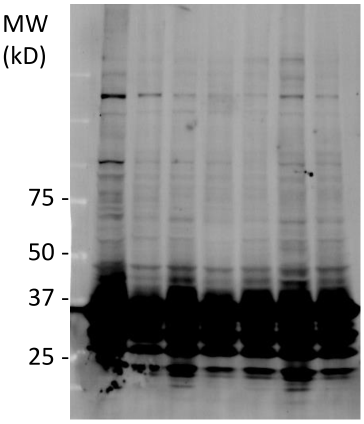

Fig. 6f

pERK

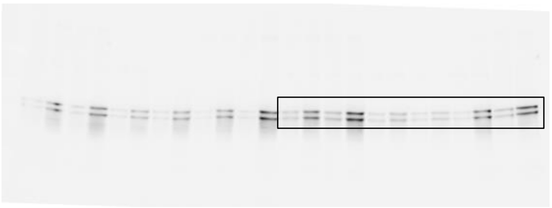

pERK long exposure

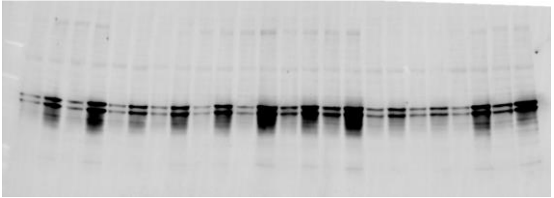

GAPDH

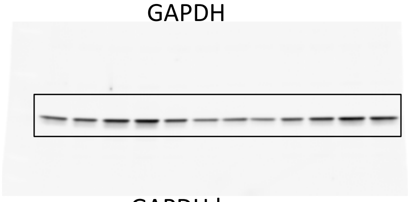

GAPDH long exposure

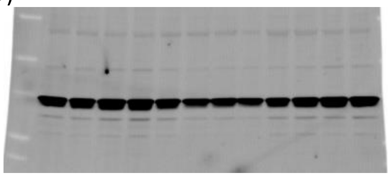

Uncropped immunoblots for Fig. 6b

pERK

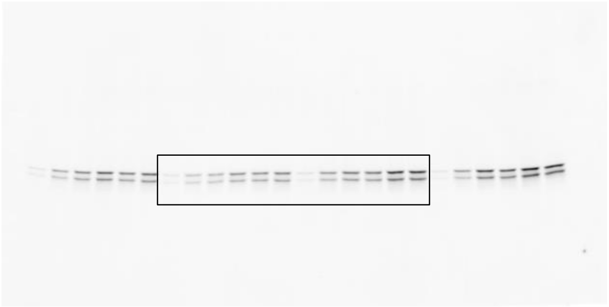

pERK long exposure

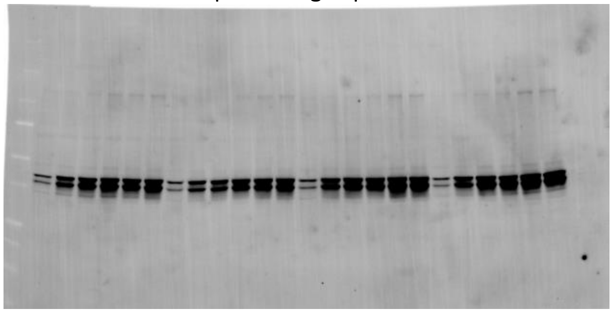

pFAK

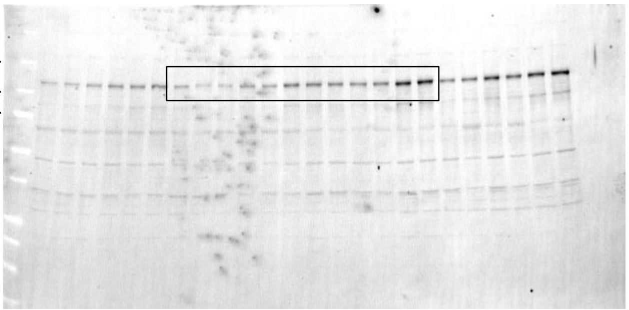

GAPDH

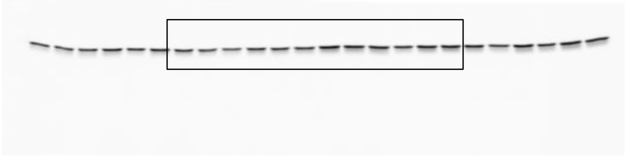

GAPDH long exposure

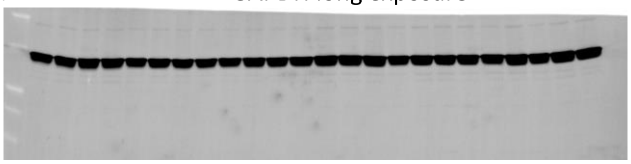

Supplementary Figure 8

Uncropped immunoblots for Supplementary Fig. 5c

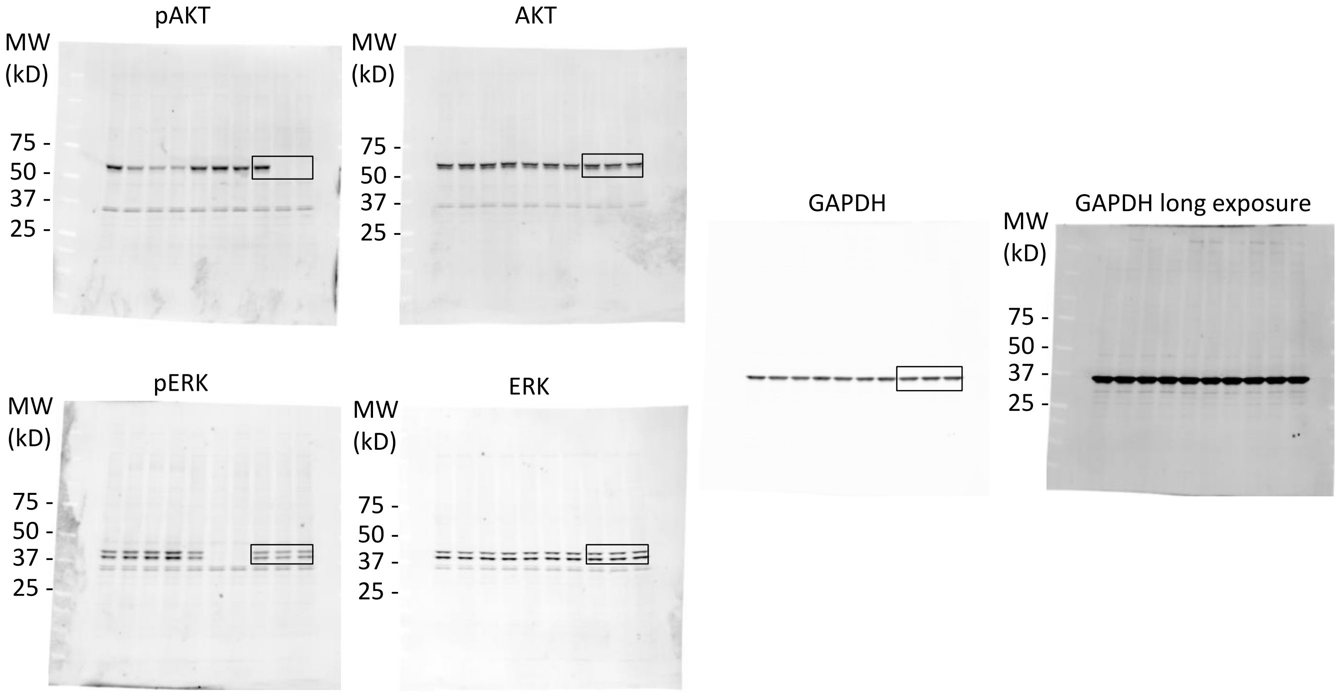

Uncropped immunoblots for Supplementary Fig. 5d

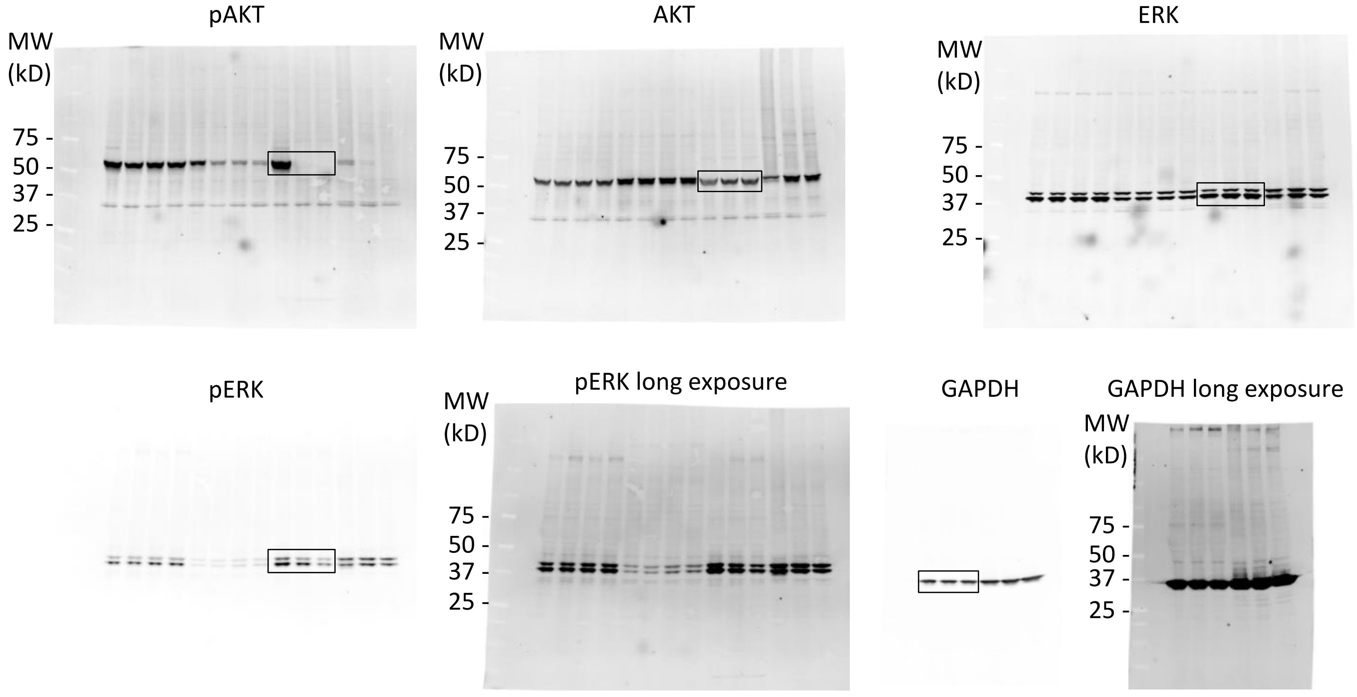

Supplementary Figure 9

Uncropped immunoblots for Supplementary Fig. 6b

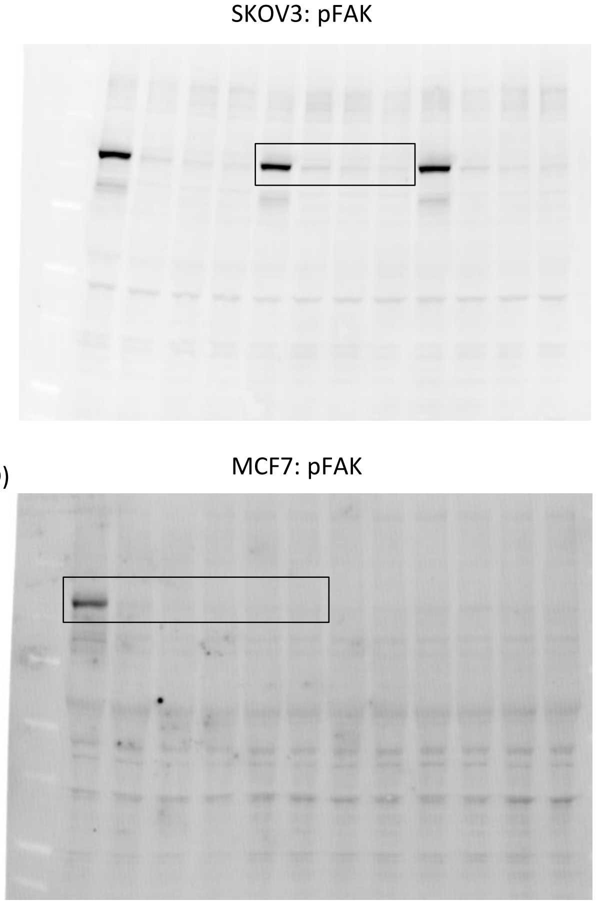

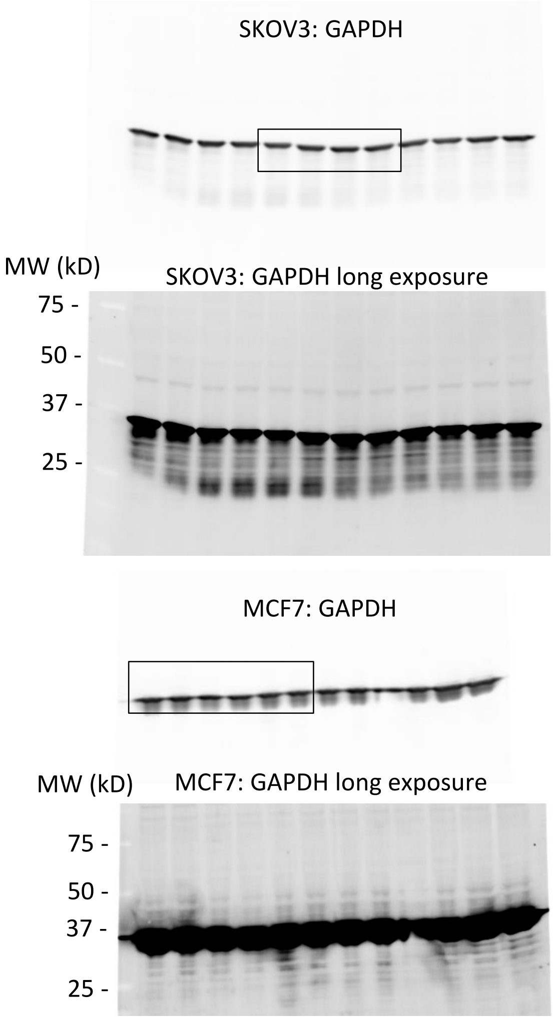

Uncropped immunoblots for Supplementary Fig. 6c, d

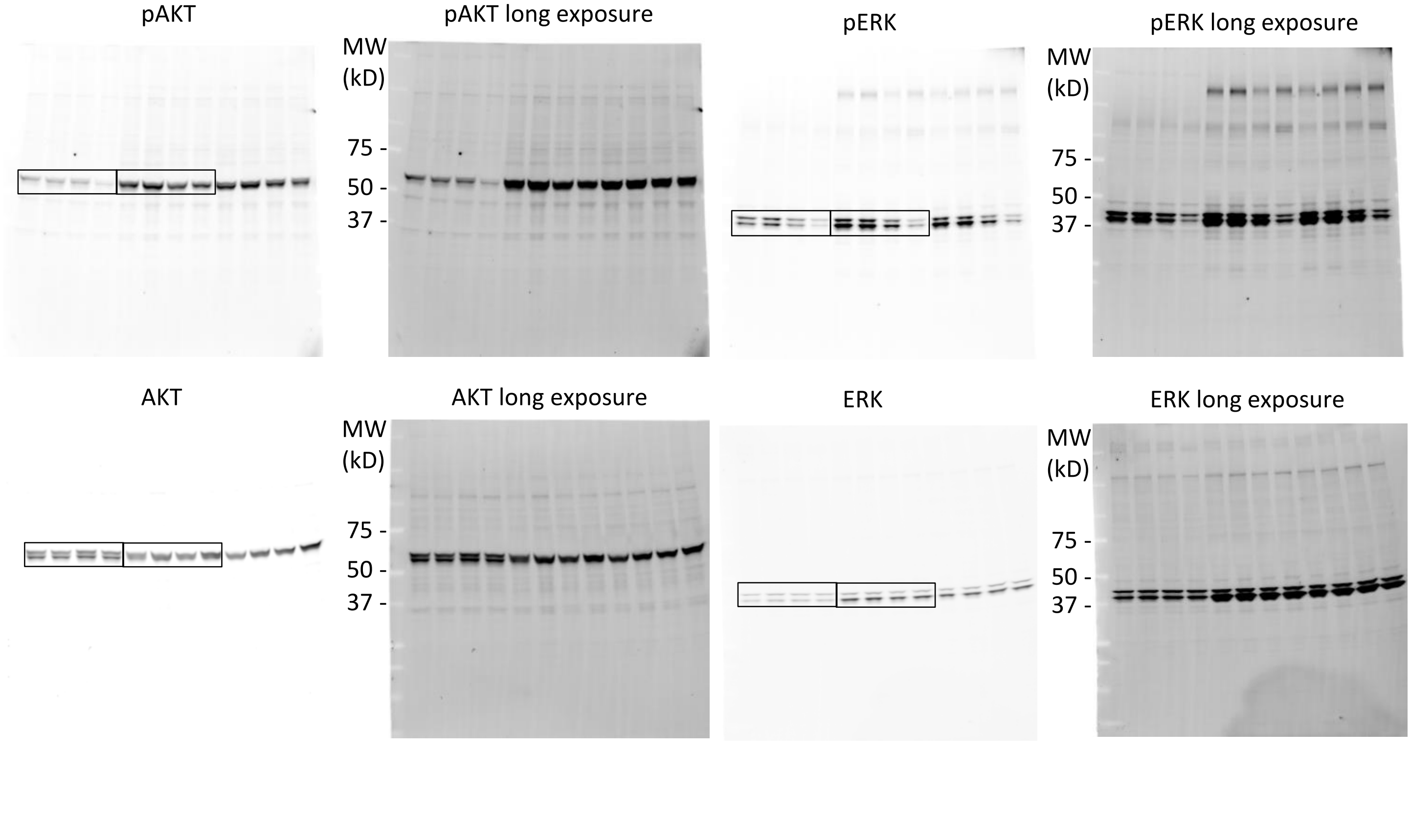

Supplementary Figure 10

Uncropped immunoblots for Supplementary Fig. 6c, d (continued)

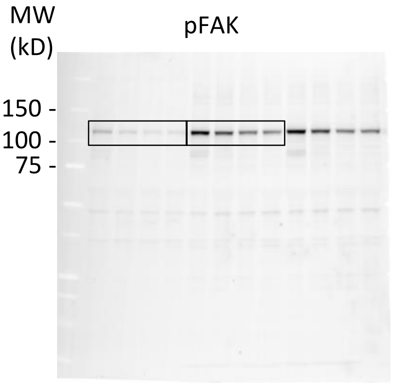

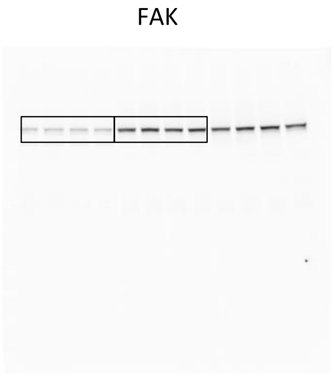

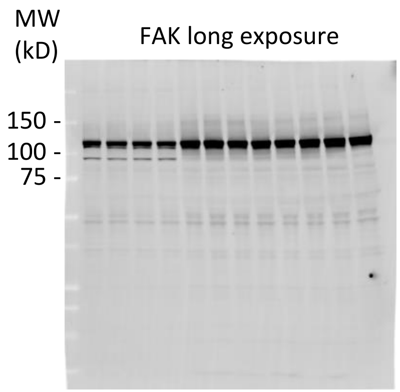

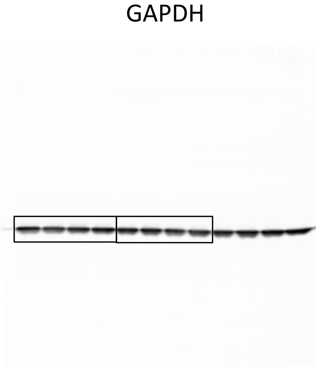

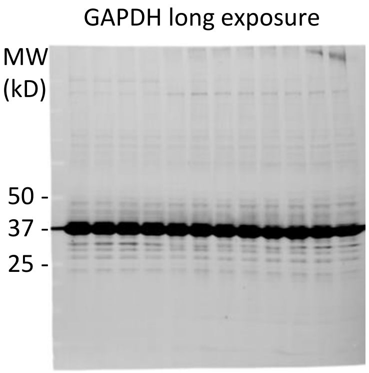

Uncropped immunoblots for Supplementary Fig. 6f

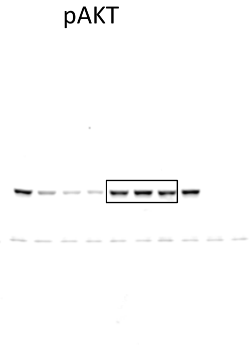

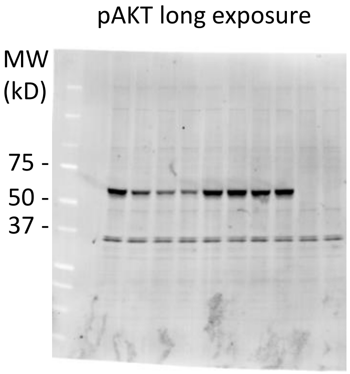

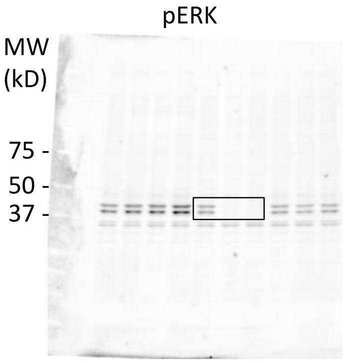

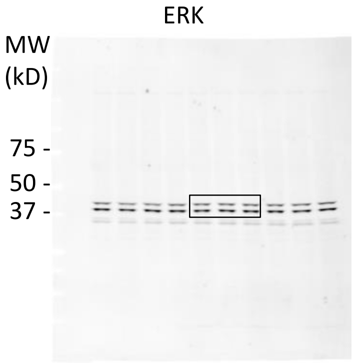

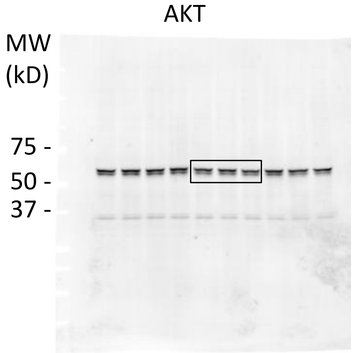

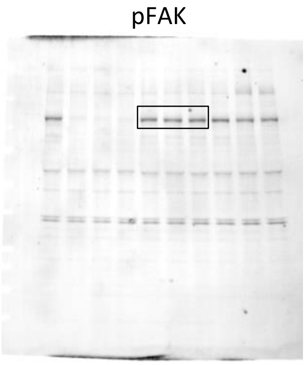

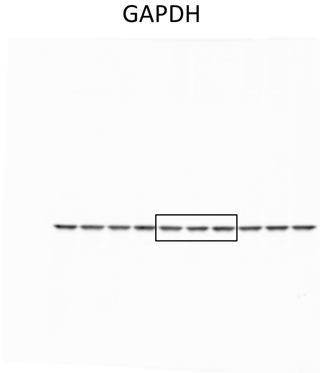

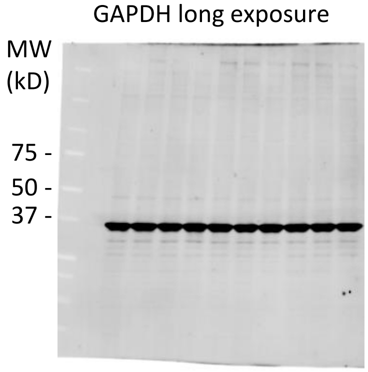

Supplementary Figure 11

Uncropped immunoblots for Supplementary Fig. 6g

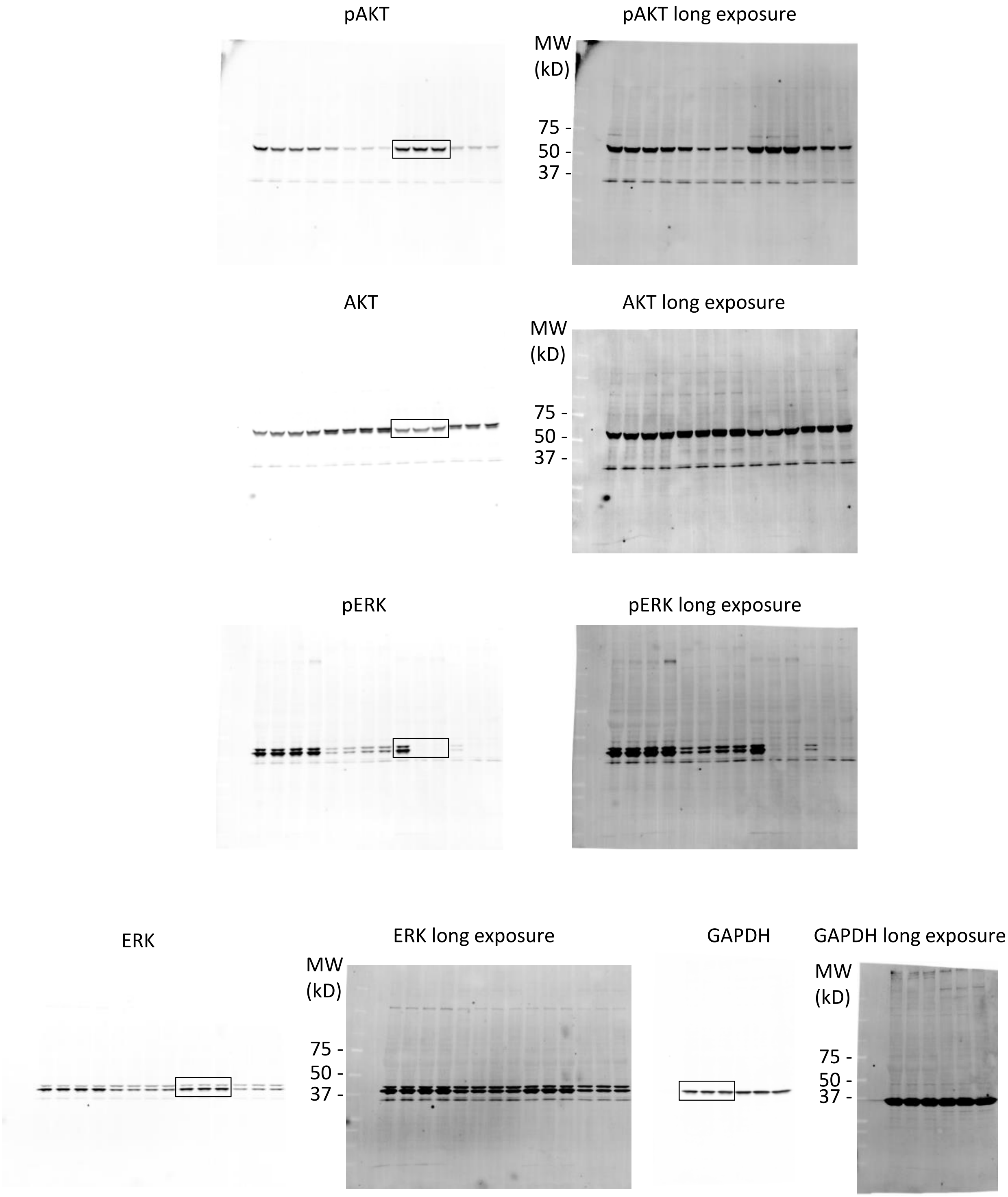

Supplementary Table 1

Supplementary Table 1. Simulation Parameters

Excitable System

a_1	0.167	b_1	45 (low activity)
a_2	16.67		41 (high activity)
a_3	167		49 (EGF)
a_4	1.2	ε	0.07
a_5	1.47	D_Y	1
D_X	1.5	R	0.5 (EGF)
U_N	0 (mean)		
	0.3 - 0.4 (std. dev.)		

Ultrasensitive System

c_1	5	c_4	0.3
c_2	50	c_5	0.02
c_3	0.01		
