# Supplementary material for: Integrating chemical and mechanical signals through dynamic coupling between cellular protrusions and pulsed ERK activation
Source: Nat Commun. 2018 Nov 7;9:4673. doi: 10.1038/s41467-018-07150-9 (PMC6220176; doi:10.1038/s41467-018-07150-9)
Supplement: Supplementary file 3 — Description of Additional Supplementary Files [file 41467_2018_7150_MOESM3_ESM.pdf]

Description of Additional Supplementary Files

Supplementary Movie 1. Ras activities on protrusions of SKOV3 cells. Time-lapse TIRF video of an SKOV3 cell expressing the Ras biosensor RBD-RFP and membrane marker Lyn-CFP imaged at 90 spf (second per frame). The video is shown at 20 fps (frame per second) and corresponds to Supplementary Fig. 1c.

Supplementary Movie 2. PI3K activities on protrusions of SKOV3 cells. Time-lapse TIRF video of an SKOV3 cell expressing the PI3K biosensor PH-AKT-RFP and the membrane marker Lyn-CFP imaged at 30 spf. The video is shown at 20 fps and corresponds to Supplementary Fig. 1d.

Supplementary Movie 3. PI3K and actin activities on protrusions of SKOV3 cells. Time-lapse TIRF video of the F-actin marker LifeAct-RFP and PH-AKT-GFP normalized to Lyn-CFP in an SKOV3 cell imaged at 90 spf. The video is shown at 20 fps and corresponds to Supplementary Fig. 1f.

Supplementary Movie 4. Coupling of ERK pulses to protrusions in MCF7 cells. Time-lapse video of an MCF7 cell expressing RBD-RFP (TIRF, top left) and ERKKTR-GFP (epifluorescence, bottom left) imaged at 90 spf. Shown on the right are FDM-processed images to highlight activities of RBD-RFP on protrusions (top) and internal flashes (bottom). The video is shown at 20 fps and corresponds to Fig. 1b, d, and Supplementary Fig. 4.

Supplementary Movie 5. LLSM images of coupling between protrusions and ERK pulses in MCF7 cells. Time-lapse LLSM video of an MCF7 expressing ERKKTR-RFP and Paxillin-GFP imaged at 60 second per volume (spv). Four different perspectives of the same cell are shown at 20 fps and correspond to Fig. 1j.

Supplementary Movie 6. Internal flashes of PI3K activities in SKOV3 cells before and after latrunculin treatment. FDM-processed time-lapse TIRF video of LifeAct, PH and Lyn for an SKOV3 cells before and after treatment with 5 uM of Latrunculin A. The cell is the same as in Supplementary Movie 3. The video is shown at 20 fps and corresponds to Supplementary Fig. 3c.

Supplementary Movie 7. LLSM images showing lack of ERK activation in an MCF7 cell making rapid protrusions not adhering to the substrate. Time-lapse LLSM video of an MCF7 expressing ERKKTR-GFP and PH-AKT-RFP imaged at 30 spv. Two different perspectives of the same cell are shown at 20 fps.

Supplementary Movie 8. Protrusion frequency analysis of 4T1, SKOV3, U2OS, HeLa, and MCF7 cells. (Left) Time-lapse TIRF video of 4T1, SKOV3, U2OS, HeLa, and MCF7 expressing PH-AKT imaged at 90 spf. (Right) Protrusions identified by FDM processing of the time-lapse video. The video is shown at 20 fps and corresponds to Fig. 2d.

Supplementary Movie 9. Sustained ERK activation driven by continuous protrusions in SKOV3 cells. Time-lapse video of an SKOV3 cell expressing PH-AKT-GFP (TIRF) and ERKKTR-RFP (epifluorescence) imaged at 180 spf. The video is shown at 10 fps and corresponds to Fig. 2g.

Supplementary Movie 10. Effect of PI3K inhibition on protrusions and ERK activity. Time-lapse video of five SKOV3 cell expressing PH-AKT-GFP (TIRF) and ERKKTR-RFP (epifluorescence) imaged at 180 spf. 1 μM of the PI3K inhibitor ZSTK474 was added at the indicated time, causing nuclear entry of ERKKTR and loss of protrusions. The video is shown at 10 fps and corresponds to Supplementary Figure 5b.

Supplementary Movie 11. Continued generation of protrusions in the presence of ERK inhibition. Time-lapse video of an SKOV3 cell expressing PH-AKT-GFP (TIRF) and ERKKTR-RFP (epifluorescence) imaged at 180 spf. 1 μM of the MEK inhibitor PD325901 was added at the indicated time, causing nuclear entry of ERKKTR but the cell continued to make protrusions. The video is shown at 20 fps.

Supplementary Movie 12. Coupling of ERK and protrusions in MCF7 cells on soft and stiff substrates. Time-lapse confocal images of MCF7 cells expressing ERKKTR on soft (0.5 kPa, top) and stiff (50 kPa, bottom) substrates imaged at 180 spf. The video is shown at 10 fps and corresponds to Fig. 6d.
