# Supplementary material for: Integrating chemical and mechanical signals through dynamic coupling between cellular protrusions and pulsed ERK activation
Source: Nat Commun. 2018 Nov 7;9:4673. doi: 10.1038/s41467-018-07150-9 (PMC6220176; doi:10.1038/s41467-018-07150-9)
Supplement: Supplementary file 16 — Reporting Summary [file 41467_2018_7150_MOESM16_ESM.pdf]

nature research

Corresponding author(s): Chuan-Hsiang Huang

Reporting Summary

Nature Research wishes to improve the reproducibility of the work that we publish. This form provides structure for consistency and transparency in reporting. For further information on Nature Research policies, see Authors & Referees and the Editorial Policy Checklist.

Statistical parameters

When statistical analyses are reported, confirm that the following items are present in the relevant location (e.g. figure legend, table legend, main text, or Methods section).

n/a	Confirmed	
☐	☒	The exact sample size (*n*) for each experimental group/condition, given as a discrete number and unit of measurement
☐	☒	An indication of whether measurements were taken from distinct samples or whether the same sample was measured repeatedly
☐	☒	The statistical test(s) used AND whether they are one- or two-sided<br>*Only common tests should be described solely by name; describe more complex techniques in the Methods section.*
☐	☒	A description of all covariates tested
☒	☐	A description of any assumptions or corrections, such as tests of normality and adjustment for multiple comparisons
☐	☒	A full description of the statistics including central tendency (e.g. means) or other basic estimates (e.g. regression coefficient) AND variation (e.g. standard deviation) or associated estimates of uncertainty (e.g. confidence intervals)
☒	☐	For null hypothesis testing, the test statistic (e.g. *F*, *t*, *r*) with confidence intervals, effect sizes, degrees of freedom and *P* value noted<br>*Give P values as exact values whenever suitable.*
☒	☐	For Bayesian analysis, information on the choice of priors and Markov chain Monte Carlo settings
☒	☐	For hierarchical and complex designs, identification of the appropriate level for tests and full reporting of outcomes
☐	☒	Estimates of effect sizes (e.g. Cohen's *d*, Pearson's *r*), indicating how they were calculated
☐	☒	Clearly defined error bars<br>*State explicitly what error bars represent (e.g. SD, SE, CI)*

Our web collection on statistics for biologists may be useful.

Software and code

Policy information about availability of computer code

Data collection	N/A
Data analysis	The following statement is included in Methods:<br>The model and all simulations are implemented using Matlab (MathWorks). The computer code is available on request.

For manuscripts utilizing custom algorithms or software that are central to the research but not yet described in published literature, software must be made available to editors/reviewers upon request. We strongly encourage code deposition in a community repository (e.g. GitHub). See the Nature Research guidelines for submitting code & software for further information.

Data

Policy information about availability of data

All manuscripts must include a data availability statement. This statement should provide the following information, where applicable:

- Accession codes, unique identifiers, or web links for publicly available datasets
- A list of figures that have associated raw data
- A description of any restrictions on data availability

The following statement is included in Methods:

All data supporting the findings of the current study are available within the Article and its Supplementary Information files or from the corresponding author upon reasonable request.

Field-specific reporting

Please select the best fit for your research. If you are not sure, read the appropriate sections before making your selection.

☒ Life sciences ☐ Behavioural & social sciences ☐ Ecological, evolutionary & environmental sciences

For a reference copy of the document with all sections, see nature.com/authors/policies/ReportingSummary-flat.pdf

Life sciences study design

All studies must disclose on these points even when the disclosure is negative.

Sample size	No statistical methods were used to determine sample sizes. However, a sample size of 3 or more was used to assess reproducibility.
Data exclusions	No data was excluded.
Replication	Reproducibility was established by at least 3 independent experiments.
Randomization	No randomization was performed since there were no animals or human participants.
Blinding	No blinding was required since there were no animals or human participants.

Reporting for specific materials, systems and methods

Materials & experimental systems

n/a	Involved in the study	
☒	☐	Unique biological materials
☐	☒	Antibodies
☐	☒	Eukaryotic cell lines
☒	☐	Palaeontology
☒	☐	Animals and other organisms
☒	☐	Human research participants

Methods

n/a	Involved in the study	
☒	☐	ChIP-seq
☒	☐	Flow cytometry
☒	☐	MRI-based neuroimaging

Antibodies

Antibodies used

The following antibodies were used for immunoblotting:
Primary antibodies were all purchased from Cell Signaling and included (cat#, species):
-Rabbit anti-phospho-ERK (#9101, human)
-Mouse anti-ERK1/2 (#9107, human),
-Rabbit anti-phospho-AKT (Ser473) (#4060, human)
-Mouse anti-AKT (#2920, human)
-Rabbit anti-phospho-FAK (Y397) (#8556, human)
-Rabbit anti-FAK (#13009, human)
-Rabbit anti-GAPDH (#2118, human).
Fluorescent Alexa 488-conjugated donkey anti-rabbit or anti-mouse antibodies (Life Technologies A21206, A21202) were used as secondary antibodies to visualize the protein bands on the Pharos Molecular Imager (BioRad).

Validation

Validation for the antibodies was provided at the Cell Signaling website (www.cellsignal.com).

Eukaryotic cell lines

Policy information about cell lines

Cell line source(s)

MCF7, SKOV3, 4T1, and HeLa were purchased from ATCC for previous studies.
U2OS was purchased from ATCC for this study.

Authentication	Cell lines were authenticated by the supplier or STR profiling using GenePrint 10.
Mycoplasma contamination	Cell lines were routinely tested for mycoplasma contamination by PCR.
Commonly misidentified lines<br>(See ICLAC register)	None was listed in the ICLAC database.
